# Quality of life and level of burden for caregivers of Indian children with post-operative congenital anorectal malformations

**DOI:** 10.6026/973206300200566

**Published:** 2024-05-31

**Authors:** Nitu Godara, Geetarani Nayak, Bikasha Bihary Tripathy

**Affiliations:** 1 College of Nursing, All India Institute of Medical Sciences, Bhubaneswar, India; 2 Department of Pediatric Surgery, All India Institute of Medical Sciences, Bhubaneswar, India

**Keywords:** Anorectal malformation, care givers, disability parenting

## Abstract

Parenting starts much before the baby is born. It always comes with mixed feelings during parenting. Therefore, it is of interest to report the quality of life and caregiver burden for Indian children with post-operative congenital anorectal malformations. We recruited total 56 caregivers for the present study. Data shows that the highest caregiver burden in economical and the lowest in psychological. Quality of life (QOL) was highest in physical domain and there was moderate negative correlation among caregiver burden, psychological health, and social relationship.

## Background:

Parenting starts much before the baby is born. It always comes with mixed feelings. This leaves the parents horrified as all of their positive's representations or manifestations come untrue [1-2]. Parents find that raising a child with a congenital defect is extremely difficult. Anorectal malformation (ARM) is one of the most prevalent congenital deformities treated in paediatric surgery. With a global frequency of 1:1500 to 1:5000, ARM is slightly more frequent in men [2, 3-4]. The urogenital, circulatory, skeletal, and digestive systems are all prone to common abnormalities [4]. Pregnancy-related environmental exposure as well as complex interactions and genetic variables can all contribute to ARM [5, 6-7]. Malformations range in severity from mild to complicated, and they can be further divided into low, moderate, and high forms. The primary treatment for ARM is surgery, with the aim of restoring sexual, digestive, and urine function [6- 7]. Simple perineal treatments can be used to treat low anorectal anomalies, but many surgical operations must be staged in order to repair high and intermediate defects. Following birth, a temporary colostomy is often necessary, with a final operation performed at a later time [7-8]. The strong relationship between the caregiver's stresses can results into chronically ill youngster [9]. The health of the carer may have an adverse effect on the child's results in terms of health, may cause difficulties in managing the child's chronic illness, and may have an adverse effect on the family as a whole [10]. Kid with anorectal malformation has a highly significant burden impact on the mother [11]. The health of the parents influences their offspring, either directly or indirectly. Nonetheless, society frequently disregards parents' psychological well-being and quality of life [5-6]. Parents do indicate a need for support and struggle to cope with the consequences of the condition. Patient care can be enhanced if assistance is customised to address these particular issues [12-13]. Therefore, it is of interest to find out whether there is an association among the caregiver burden and quality of life in caregiver, in children with post-operative congenital anorectal malformations.

## Materials and Method:

In this study, a non-experimental quantitative approach was used. The purpose of the study was to assess the burden on caregiver and quality of life in caregiver of children with postoperative congenital anorectal malformations. Variables are the determinable traits of an idea and it involves a reasonable group of attributes. The research variables are quality of life and caregiver burden. The research was carried out at the paediatric surgery outpatient department of AIIMS Bhubaneswar. In one year around 35 patients of anorectal malformation get operated in AIIMS, Bhubaneswar and come for follow up in the Paediatric Surgery OPD. Total 56 participants were included after considering the inclusion and exclusion criteria. The Population selected for the study comprised caregivers of children with post-operative congenital anorectal malformations. The demographic Performa consists of child and care givers details. The CBS-IP scale was used to assess the caregiver burden in caregiver of children with postoperative congenital anorectal malformations, which was a standard tool.

The tool evaluated the QOL tool has 4 domains: physical (5 items), economical (5 items), social (5 items), and psychological (5 items). There were a total 20 items. It was four-point Likert scales from 1 to 4

The burden scoring criteria:

[1] 0-25 =Absent or little

[2] 26-50 = Mild

[3] 51-75 = Moderate

[4] 76-100=Severe

The score was classified into absent or little, mild, moderate and severe burden. The score ranges from 0-100 points.

## Tool WHOQOL-BREF in various domains:

A standard technique for assessing the quality of life of carers of children with postoperative congenital anorectal malformation is the WHOQOL BREF scale. The 26-item tool is intended to gauge one's quality of life. The items are graded on a 5-point scale, providing a profile with two individually scored items representing an individual's overall opinion of quality of life and health (Q1 and Q2) and four domain scores (physical health, psychological health, social relationship, and environment). With a score range of 0-100, the four domains are ranked positively, with a higher score indicating a higher quality of life.The validity of the socio- demographic proforma for caregiver and child scale was obtained from 5 experts. The content validity of the Caregiver burden scale Indian Population scale was already established as it was a standardized tool. The validity of the WHOQOL-BREF scale was already established as it was a standardized tool.

## Data collection method:

The project was started after getting permission from the institutional ethical research committee and approval from the principal College of Nursing and medical superintend of AIIMS, Bhubaneswar. The entire research process was explained to the participants and written consent was obtained from them via a Participant information sheet (PIS) prior to data collection. The collected data were arranged, coded, analyzed and interpreted. Descriptive and inferential statistics was used for data analysis.

## Results:

Parameters used in descriptive statistics were frequency, percentage, median, range; and parameters used in inferential statistics are square and Spearman rank correlation test. The normality of the data was evaluated using the Shapiro- Wilk test at the level of significance of 0.05. The data found non-normally distribution as the p value <0.05.

Table 1 shows that, 58.9% children were male. Majority of (46.4%) children were between 1-3 years of age group. Nearly half of the children (44.6%) were diagnosed immediately after birth and 57.1 % children had associated another anomaly with anorectal malformation. More than one fourth (33.9%) children had incontinence after surgery during follow up.

Table 2 depicted that more than half of the caregiver (69%) had moderate burden. Only 7% of caregiver had severe burden. Nearly one fourth of them (25%) had mild burden.

Table 3 shows the highest caregiver burden was there in economic and lowest were in psychological. The mean economic score and psychological score were 94.10 ± 32.36 and 45.09 ± 22.57 respectively.

Table 4 shows the social domain QOL was highest and in physical, QOL was poor. The mean physical, psychological, Social, Environment score QOL score were 52.61±19.44, 54.75±24.89, 58.58±30.80, 51.82±15.03 respectively.

As depicted in Table 5, there was moderate negative correlation between caregiver burden and psychological health (p=0.002 & r= -0.31) and also there is a moderate negative correlation between caregiver burden and social relationships (p=0.001& r= -0.38) As depicted in Table 6, the association between caregiver burden and type of surgery which was statistically significant (χ^2^ = 22.62, p <0.001). Association between caregiver burden and associated other anomalies with ARM was not statistically significant. (χ^2^ = 3.03, p <0.26).

Data presented in Table 7, shows that there was no statistically significant association between caregiver burden and age group of children. (χ^2^=1.336, p < 0.85).

Data presented in table no-8 shows that there was statistically significant difference in quality of life (physical) between the group having other associate anomaly and those who don't have as (t=-2.05, p =0.04)

Table 9 depicts that there is no difference in domain wise quality of life between one stage repair and three stage repair surgery. (p <0.05)

## Discussion:

Common congenital anomalies with a wide range of problems are anorectal malformations. Simple perineal treatments can be used to manage low anorectal anomalies, but numerous surgical operations must be performed to rectify high and intermediate defects [1, 2]. We observed that more than half of the caregiver (69%) had moderate burden. Nearly one fourth of them (25%) had mild burden. Only 7% of caregiver had severe burden. In association to our findings, Ahmadi *et al.* found that the level of caregiver burden as severe (4.4%), moderate to severe (21.1%), mild to moderate (40%) and 17.6% had low burden, 71.2% had moderate burden, 11.2% has severe burden [14]. In contrast to our findings Arab *et al.* reports that more than half (57%) having severe burden [15]. Valença *et al.* found a moderate and severe burden levels among 43% of sample [16]. This may be due to the reason that difference in sample size, because of low socioeconomic status of families. In present research; the highest caregiver burden was in economic domain and lowest were in psychological domain. The mean physical score was 56.16± 22.64. The mean economic score was 94.10 ± 32.36. The mean time score was 60.89± 21.87. The mean social score was 50.32 ± 20.34. Present study finding revealed that QOL in social domain score of caregivers of children with post-operative congenital anorectal malformations was highest and in physical domain QOL was very poor.

In contrast to present data, Razera *et al.* revealed that the mean score of the social for quality of life of caregivers was lowest (9.33±1.49), followed by psychological (18.23±1.50). The physical and psychological subgroups scores were statistically higher in primary caregiver [17].We found that, there was moderate negative correlation between caregiver burden and psychological health and moderate negative correlation between caregiver burden and social relationships (p=0.001 & r=-0.38). Temple *et al.* concluded that, there was moderate negative correlation between caregiver burden and psychological health (p=0.003 & r=-0.21) and moderate negative correlation between caregiver burden and social relationships (p=0.004 & r=-0.42) [18]. We found no statistically considerable association between caregiver burden and age group of children. (χ^2^=1.336, p < 0.85). It is in association with Gbolahan *et al.* findings [19]. This could be due to more number of children in 0-2 months and burden for these children is more compared with other age group. We found that there is no diversity in domain wise quality of life between one stage repair and three stage repair surgery(p <0.05). The reason behind it is; no difference is the type of ARM, less hospital stays and good cosmetic surgery. This finding contradicts Sadighian *et al.* research, which reported the difference in quality-of-life domain. Quality of life score was highest in psychological domain and least in environment domain. The reason is good family environment in developed country and lack of social support in nuclear family and busy life style [20].

## Implication:

The present study got implications for nursing education, nursing practice, nursing research, and nursing administration.

[1] Nursing student should be trained regarding provision of care of children suffering from anorectal malformation and further training should be provided in management of Anorectal malformation.

[2] A nurse's provision of suitable assistance to parents can enhance their self-assurance in life, preserve their mental health, act as a buffer against the daily care and carer load, and enhance parent-child relationships.

[3] Nursing administration must provide separate counselling session to the caregiver whose children are suffering Anorectal malformation and further training should be provided in management of anorectal malformation.

## Limitations:

[1] It is a single centre study.

[2] The heterogeneity among the various types of anorectal malformations.

[3] Sample size was less for the study that could affect the generalizability.

## Recommendation:

[1] Comparative study can be conducted among caregiver of children with postoperative High, Intermediate and low ARM.

[2] Qualitative study can be conducted among caregiver related to caregiver burden and quality of life.

[3] Similar study can be conducted by taking large samples involving other tertiary care hospital all over India.

## Conclusion:

Data shows that the highest caregiver burden is economical and the lowest is psychological. Quality of life (QOL) was highest in physical domain and there was moderate negative correlation among caregiver burden, psychological health, and social relationship.

## Figures and Tables

**Table 1:** Frequency and percentage of socio-demographic characteristics of Children with congenital anomalies (n = 56)

**Demographic Variables**		**Frequency**	**Percentage**
Gender	Male	33	58.9
	Female	23	41.1
Age group	0 - 1 years	10	17.9
	1 - 3 years	26	46.4
	3 -5 years	20	35.7
Age at diagnosis	Immediately after birth	25	44.6
	Within 24 hours of birth	19	33.9
	Between 24 to 48 hours of birth	12	21.4
Type of anorectal malformation	High ARM	28	50
	Low ARM	28	50
Congenital anomaly	Only anorectal malformation	24	42.9
	Associate another anomaly with ARM	32	57.1
Type of surgery	Single stage Repair	28	50
	3 stage Repair	28	50
Complication after surgery/during follow up	Re-do surgery	4	7.1
	Anal stenosis	2	3.6
	Incontinence	19	33.9
	Constipation	6	10.7
	Recurrent Urinary Tract Infection	14	25
	More than one complication	11	19.6
Family Support	Yes	12	21.4
	No	44	78.6

**Table 2:** Frequency and percentage distribution of level of caregiver burden in caregiver of children with post-operative congenital anorectal malformations (n=56)

**Variables**		**Frequency**	**Percentage**
Caregiver Burden	Mild Burden	14	25
	Moderate Burden	38	67.9
	Severe Burden	4	7.1

**Table 3:** Domain wise Caregiver burden score of caregivers of children with post-operative congenital anorectal malformations (n=56)

**Variables**		**Mean ± Standard Deviation**
Caregiver Burden	Physical	56.16± 22.64
	Economic	94.10 ± 32.36
	Time	60.89± 21.87
	Social	50.32 ± 20.34
	Psychological	45.09 ± 22.57

**Table 4:** Domain wise quality of life score of caregivers of children with post-operative congenital anorectal malformations (n = 56)

**Variables**		**Mean ± Standard Deviation**
Quality of Life	Physical	52.61±19.44
	Psychological	54.75±24.89
	Social	58.58±30.80
	Environment	51.82±15.03

**Table 5:** Relationship between caregiver burden and Quality of life of the caregivers (n=56)

**Variables**		**Test statistics**	
		**P**	**"P" Value<0.05**
Caregiver Burden	Physical QOL	-0.19	0.15
	Psychological Health QOL	-0.31	0.002*
	Social Relationships QOL	-0.38	0.001*
	Environment QOL	-0.22	0.09

**Table 6:** Association between caregiver burden and different variables n=56

**Caregiver Burden**		**Mild**	**Moderate**	**Severe**	**DF**	**χ^2^**	**P value**
Type of Surgery	Single Stage Repair	14	14	0	2	22.6	<0.001**
	3- Stage Repair	0	24	4			
Associated Anomalies	Yes	8	20	4	2	3.03	0.26
	No	6	18	0			

**Table 7:** Association between caregiver burden and age group of children (n=56)

**Age Group**	**Mild Burden**	**Moderate Burden**	**Severe Burden**	**χ^2^**	**P value**
0-1Years	3	7	0		
1-3 years	7	17	2	1.336	0.85
3-5 years	4	14	2		

**Table 8:** Comparison of domain wise quality of life between presence and absence of other associated anomalies (n=56)

	**Group**			
Variables (QOL Domain)	With associated Anomaly (mean ± SD)	Without associated Anomaly (mean ± SD)	t- Value	p -Value
Physical	16.5 ± 4.11	21.88 ± 4.37	2.05	0.04
Psychology	12.82 ± 3.15	20.80 ± 3.87	0.652	0.64
Social relationship	10.10 ± 1.01	10.22 ± 1.54	0.724	0.75
Environment	20.64 ± 3.63	79.08 ± 11.16	0.735	0.67

**Table 9:** Comparison between Quality of life and type of surgery done in children (n=56)

	**Group**			
Variables(QOL Domain)	Single Stage Repair (mean ± SD)	Three stages Repair (mean ± SD)	t- value	p -Value
Physical	35.94 ± 6.61	33.84 ± 7.19	0.786	0.69
Psychology	34.78 ± 6.79	34.12 ± 8.33	0.708	0.72
Social relationship	25.62 ± 11.30	26.78 ± 7.73	0.743	0.63
Environment	34.78 ± 6.79	34.12 ± 8.33	0.734	0.72
